# Early treatment of open diaphyseal tibia fracture with intramedullary nail versus external fixator in Tanzania: Cost effectiveness analysis using preliminary data from Muhimbili Orthopaedic Institute

**DOI:** 10.1051/sicotj/2019022

**Published:** 2019-06-17

**Authors:** Billy T. Haonga, Mapuor M.M. Areu, Sravya T. Challa, Max B. Liu, Edmund Elieza, Saam Morshed, David Shearer

**Affiliations:** 1 Department of Orthopaedic and Traumatology, Muhimbili University of Health and Allied Sciences Kalenga street 11000 Dar es Salaam Tanzania; 2 Department of Orthopaedic Surgery, University of California San Francisco 2550 23rd Street San Francisco CA 94110 USA

**Keywords:** Tibial fracture, Cost-effectiveness, Intramedullary nail, Cost of treatment

## Abstract

*Introduction*: Open tibia fractures are some of the most common types of Orthopedics injuries in low- and middle-income countries (LMICs). In Tanzania, open tibia fractures are treated either conservatively by prolonged cast or surgically by external fixation (EF) or intramedullary nail (IMN) when available. The cost of treatment and amount of time patients spend away from work are major economic concerns with prolonged casting and EF. The goal of this study was to determine the cost effectiveness of IMN versus EF in the treatment of open diaphyseal tibia fractures at Muhimbili Orthopaedic Institute (Dar es Salaam, Tanzania).

*Methods*: This is a prospective randomized control study conducted of patients with a closeable AO/OTA 42 open diaphyseal tibia fracture. The patients underwent surgical fixation with either IMN or EF at Muhimbili Orthopaedic Institute (MOI), and were followed up at 2, 6, and 12 weeks postoperatively. A micro-costing method was used to estimate the fixed and variable costs of IMN and EF of the open diaphyseal tibial fracture.

*Results*: The mean total cost per patient was lower for the IMN group ($425.8 ± 38.4) compared to the EF group ($559.6 ± 70.5, *p* < 0.001), with savings of $133.80 per patient for the IMN group. The mean hospital stay was 2.72 ± 1.40 days for the IMN group and 2.44 ± 1.47 days for the EF group (*p* = 0.5). Quality-adjusted life years (QALYs) were 0.26 per patient for the IMN group and 0.24 in the EF group at 12 weeks (*p* = 0.8). Ninety-two percent of patients in the IMN group achieved fracture union versus 60% in the EF group at three months postoperatively (*p* = 0.03).

*Conclusion*: IM nailing of a closeable open diaphyseal tibial fracture is more cost effective than EF. In addition, IM nailing has better union rates at three months compared to EF.

## Introduction

Cost effectiveness analysis has become a valuable tool in public health as it aids decision-makers in identifying the most effective ways of allocating resources for the prevention, diagnosis, and treatment of diseases. It has been used to compare different treatment modalities in various medical and surgical fields [1, 2]. An intervention is said to be more cost effective if it is:

- Less costly with an equal or better outcome (preferred treatment) [2, 3],
- Less costly with a worse outcome (benefit of alternative not worth the cost),
- More costly with better outcome (benefit is worth the cost).

Africa is estimated to have the highest proportion of disability-adjusted life years (years of life lost as a result of disability) due to surgical conditions at 38 per 1000 populations [4, 5].

The tibia is the most common site of long bone fracture in high energy injuries and the care of these fractures is complicated due to lack of soft tissue coverage on the antero-medial surface [6, 7]. Approximately two tibia shaft fractures per 1000 individuals occur each year [8, 9]. In both children and adults, open tibia fractures are treated with surgical debridement followed by casting with plaster of Paris with window or fixating with external fixation (EF) or with the intramedullary nail (IMN). The introduction of IMN in the treatment of displaced diaphyseal fractures in adults has become the gold standard compared to other implants [10]. Currently, only a few centers in low- and middle-income countries (LMICs) are able to offer IMN. This is due to a number of factors including lack of technical training, lack of studies done to show its efficacy, and greater need, in certain cases, for more advanced operating equipment.

There are currently no data evaluating the cost effectiveness of treating open tibia shaft fractures surgically in LMICs designated by the World Bank. The objective of this study was to determine the cost effectiveness of IMN compared to EF in open diaphyseal tibial fractures in Tanzania.

## Methods

This was a cost analysis comparing two types of treatment for open tibial fractures using data from a separate prospective randomized trial conducted at Muhimbili Orthopaedic Institute (MOI) in Dar es Salaam, Tanzania. Institutional review board approval was obtained from Muhimbili University of Health and Allied Sciences (MUHAS).

The study was conducted from July 2016 to March 2017. Patients who have reached skeletal maturity (confirmed by X-ray) and presenting with an AO/OTA type 42 open diaphyseal tibial fracture in which the skin could be primarily closed were included in the study.

Exclusion criteria were as follows:

- Presented more than 24 h after injury,
- Pathologic fracture,
- Deformity or previous fracture of the same limb,
- Bilateral fractures,
- Ipsilateral comminuted femur fracture,
- Vascular or neurologic injury (Glasgow Coma Scale [GCS] < 12).

Eligible patients who provided informed consent were enrolled in the study. Antibiotics were provided upon presentation. Demographic data, baseline clinical information, and injury characteristics were assessed and recorded. Randomization was done immediately following intraoperative confirmation that the wound was primarily closeable. Patients were randomized to either the IM nail (SIGN Fracture Care International, Richmond, WA) or single bar external fixator (Samay Surgical, Gujarat, India). The randomization was performed using a block randomization algorithm produced by a random number generator. Patients remained in the ward for three days postoperatively, or until they were clinically fit to be discharged.

Patients were followed up at 2, 6, and 12 weeks. At each visit, patients were assessed using the EuroQol EQ-5D survey, a validated and widely used questionnaire. This survey involves a series of five questions that assess a patient’s health-related quality of life. In this study, it was used to estimate Quality-adjusted Life Years (QALYs) which take into account both a patient’s quantity and quality of life. Additionally, clinical and radiologic evidence at that three-month follow-up were used to designate the fracture as have achieved union, delayed union, or non-union. Clinical union was defined as absence of movement at the fracture site. Radiographic union was based on calcified bridging callus formation. EF patients were evaluated at three-months to determine whether the external fixator should be removed. If there was a concern for delayed union or non-union, removal of the external fixator was delayed.

Cost of treatment was determined by measuring variable direct costs (medical personnel, implants, single-use supplies, medications, consultations, X-rays, blood-tests, hospital bed) and fixed direct costs (instrument sets, ancillary staff). Medical personnel costs were estimated using time and motion analysis. At each stage of treatment, the type of medical personnel and length of time that the medical personnel spent treating a patient was recorded. Hospital accounting records were then obtained in order to calculate the average salary earned by each type of medical personnel. From this average salary figure, an estimated cost per hour was obtained; the amount of time a medical professional spent with a patient was then multiplied by the estimated cost per hour in order to obtain the average medical personnel cost per patient.

The cost of each intramedullary nail implant was estimated by adding the manufacturing costs (provided by SIGN) to the cost of shipment. The cost of external fixators and consumable supplies was obtained directly from the MOI data system. Cost of removing external fixators was incorporated in the outpatient follow-up costs. Administrative costs were estimated using the patient day equivalents (PDE) method. PDEs were calculated by taking the total number of MOI inpatients per year divided by the average length of stay. The administrative cost for open tibia fractures was then calculated by multiplying the total administrative cost by the ratio of PDEs for open tibia fractures to the total PDEs. All costs are reported in United States Dollars (USD).

Data were recorded on REDCAP (Vanderbilty University, Nashville, TN) hosted by servers at the University of California, San Francisco (San Francisco, CA). Data were analyzed using Statistical Package for Social Scientists (SPSS) 20 (IBM, North Castle, NY). Student’s *t*-test and Fischer’s exact test were used to compare the outcome of the treatment arms.

## Results

**Figure 1:**
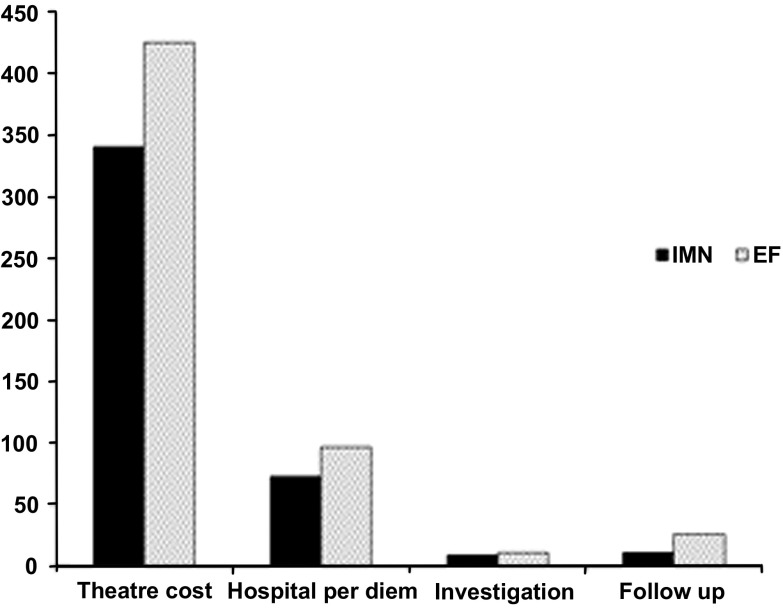
Mean total theater cost, hospital per diem, investigation and follow-up costs in the IMN and EF groups.

Fifty eligible patients were enrolled into the study, with a mean age of 32.6 years (range 20–62) for the IMN group and 32.8 (18–61) for the EF group (Table 1). During the enrollment of patients for the cost effectiveness portion of this study, the following patients were excluded: non-OTA 42 fracture (17), presentation beyond 24 h (1), skeletal immaturity (1), pathologic fracture (1), comminuted femur fracture (5), traumatic brain injury (4), and prior ipsilateral injury (1). All patients who were eligible for the study provided consent for enrollment in the study. There was no loss to follow up. The majority of the affected patients were between 18 and 39 years of age, with a male-to-female ratio of 4:1. The most common mechanism of injury was road traffic accident, accounting for 92 and 84% in IMN and EF-treated patients, respectively. Transverse and comminuted fractures were the most common fracture patterns seen in both groups. The mean total cost for the treatment of open diaphyseal tibial fracture with IMN was $425.8 ± 38.4 per patient. The total operating theater cost contributed to 79.3% of the average total cost of treatment; this figure included medical personnel ($187.0 ± 2.4), implants ($95.0), instrument sets ($7.5), medications (anesthesia, antibiotic, analgesia, infusion) ($13.2,) single-use supplies ($9.3 ± 1.0), and administrative and supportive staff ($28.2). The operating theater sterile instrument sets, the implant, and the single-use supply costs were the same for all patients, but the medical personnel and anesthesia costs were variable. The hospital per diem rate contributed to 16.2% of the average total cost of treatment, including nursing care, consultation fees, bed, meals, medications, blood work and X-rays. Costs associated with follow-up with patients for three months contributed to 2.5% of the average total cost, including outpatient consultation, nursing care, investigation, and medication (Figure 1, Table 2).

**Table 1:** Demographic data.

Demographic data	IMN	EF
Total number of patients	25	25
Male	17 (68%)	22 (88%)
Female	8 (32%)	3 (12%) (*p* = 0.03)
Mean age	32.56 (20–62)	32.8 (18–61)
Mechanism of injury		
Road traffic crash	23 (92%)	21 (84%)
Fall from height	1 (4%)	2 (8%)
Assaults	1 (4%)	1 (4%)
Heavy object fall on the leg	0 (0%)	1 (4%)
Pattern of fracture		
Transverse	11 (44%)	10 (40%)
Comminuted	9 (36%)	8 (32%)
Oblique	3 (12%)	5 (20%)
Spiral	2 (8%)	2 (8%)

EF, external fixation; IMN, intramedullary nail.

**Table 2:** Mean total (variable and fixed) costs in the IMN and EF groups.

Cost category	IMN	EF
Theatre costs	79.3%	76.0%
Medical personnel	$187.0 (±2.4)	$242.5
Implant	$95.0	$104.5
Tibia instrument set	$7.5	$10.5
Medication	$13.2	$17.8
Single-use supplies	$9.3(±1.0)	$14.7
Administrative and supportive staff	$28.2	$35.0
Hospital per diem costs	16.2%	17.5%
Ward personnel	$37.4 (±1.8)	$50.1
Bed and feeding	$26.7 (±2.3)	$36.0 (±1.1)
Consultation	$8.9	$11.0
Follow-up costs	2.5%	4.5%
Consultation	$7.5	$14.0
Nursing care	$3.3 (±0.2)	$8.0
Medication	$0.0	$4.2
Investigation costs	2%	2%
X-ray	$5.3	$6.9
Blood test	$3.2	$4.3
Operation time (min)	97.5 ± 8.3	74.6 ± 5.1
Mean length of hospital stay (days)	2.27 ± 1.4	2.44 ± 1.47
Total mean cost	425.8 (±38)	559.6 (±70.48)

EF, external fixation; IMN, intramedullary nail.

The average total cost for the treatment of open diaphyseal tibia fracture using EF was $559.6 ± 70.48 per patient. The operating room cost and the hospital per diem were almost the same for all patients in the EF group. Ward personnel, bed and food costs were higher due to repeat hospital visits or readmission for pin tract infections as well as nursing care compared to the IMN group. The cost of an extra visit required for the EF group for wound care and the cost of casting increased the three-month follow-up cost from 2.5% for the IMN to 4.5% for external fixation groups (Figure 1, Table 2).

Mean total cost was significantly higher in the EF group ($559.6 ± 70.48) compared to the IMN group ($425.8 ± 38.4, *p* < 0.001). At the three-month follow up, QALYs for the IMN and EF groups were 0.26 and 0.24 (*p* = 0.8), respectively. Treatment times for IMN and EF were 97.5 ± 8.3 min and 74.6 ± 51 min, respectively (*p* = 0.8). There was no significant difference between the mean length of hospital (LOS) for the IMN group (2.72 ± 1.4 days) and the EF group (2.44 ± 1.47 days).

**Figure 2:**
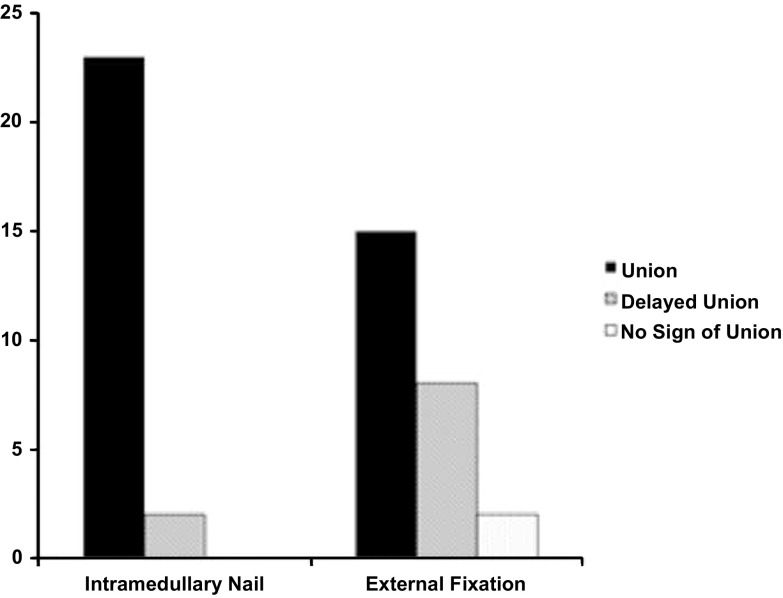
Progress to fracture union at the three month follow-up visit in the IMN and EF groups.

At the three-month follow up, 92% patients in the IMN group and 60% in the EF group showed clinical and radiologic evidence of union (*p* = 0.03). Two (8%) patients had no signs of union in the EF group while 32% had delayed union (*p* = 0.04). In the IMN group, all patients showed signs of union and 8% of patients had delayed union (*p* = 0.05). The IMN group had a better rate of union overall compared to the EF group (*p* = 0.026) (Table 3, Figure 2).

**Table 3:** Progress to union at three months postoperatively in the IMN and EF groups.

Treatment	Union	Delayed union	Nonunion
IMN	23 (92%)	2 (8%)	0 (0%)
EF	15 (60%)	8 (32%)	2 (8%)
*p*-value	0.03	0.04	0.05

EF, external fixation; IMN, intramedullary nail.

## Discussion

In 50 patients with open diaphyseal tibial fractures, our results showed that treatment with IMN is more cost-effective and has a better rate of union at three months. It is important to note that this study was conducted at an orthopedic training institute, with senior residents as principal surgeons for most of the patients. Previous literature shows that with training and experience using the SIGN IM nail, operating time decreases substantially, to 60–90 min as reported in a previous study [11]. Therefore, the operative costs associated with IMN should be expected to decrease with experience and training. At the same time, the cost of intramedullary nailing reported in this study is likely a slight underestimate because the nail implants do not as of yet have a set retail value and their costs had to be approximated by adding the manufacturing costs to the shipping costs.

Previous studies at the same institution in Tanzania have shown that femoral nailing with the same SIGN IM nail is a more cost-effective treatment compared to traction based on cost of treatment, as have studies using the same nail in Kenya and Cambodia [3, 12, 13]. While at a different anatomic location, our results support these studies in the use of IMN as a cost effective surgical management modality that should not be hindered by limited availability.

In addition to being cost-effective, the IMN approach to open diaphyseal tibial fractures also has a better union rate than EF. Previous studies have found various complications with EF ranging from delayed union to pin loosening and mal-union [14, 15]. Our results support these findings and encourage favoring IMN over EF in the management of these fractures.

This study has several limitations. From the cost perspective, we recognize that not accounting for the costs of training a surgeon or other operating room staff to learn how to place intramedullary nails versus external fixators is a limitation of this study.

Additionally, there was no standardized physiotherapy schedule for patients in either treatment arm. This was done primarily because patients came to the study center from different cities across Tanzania for treatment; it would not have been possible to ensure that patients received the same quality of physiotherapy once they returned home after their surgery. This limits the study both in terms of the clinical outcomes as well as from a cost perspective, since the costs of physiotherapy were not included.

While the study was adequately powered, the small sample size and short follow-up period of 12 weeks prevented a comprehensive comparison of the two modalities. It is not possible to definitively state what the long-term relative cost effectiveness of each modality is beyond the 12 week follow-up period. Additionally, pre-hospital treatment costs, costs of electricity and water supply, and costs from loss of productivity were not included in the study. There were also certain clinical features that were not recorded as part of the study that may contribute to the cost effectiveness comparison such as infection or reoperation rates. Future studies with more patients and a longer follow-up period are underway to determine the overall cost over a one-year period of treatment of diaphyseal tibial fractures using IMN versus EF.

Like many other developing countries, trauma remains a major public health problem in Tanzania and is a leading cause of morbidity and mortality especially among previously healthy, productive adults [16, 17]. Tibial fractures are a common consequence of trauma and are often not managed optimally in LMICs due to limited resources. Further investigation is required to strengthen the data from this study and provide more insights into the true outcomes and costs of using IMN versus EF for tibial fractures.

## Conclusion

Intramedullary nailing of a closeable open tibial fracture was found to have higher rates of union and be more cost effective than external fixation at three months.
